# The construction and visualization of the transmission networks for COVID-19: A potential solution for contact tracing and assessments of epidemics

**DOI:** 10.1038/s41598-021-87802-x

**Published:** 2021-04-21

**Authors:** Caiying Luo, Yue Ma, Pei Jiang, Tao Zhang, Fei Yin

**Affiliations:** West China School of Public Health and West China Fourth Hospital, Sichuan University, Chengdu, China

**Keywords:** Diseases, Infectious diseases, Health care, Disease prevention

## Abstract

The WHO has described coronavirus disease 2019 (COVID-19) as a pandemic due to the speed and scale of its transmission. Without effective interventions, the rapidly increasing number of COVID-19 cases would greatly increase the burden of clinical treatments. Identifying the transmission sources and pathways is of vital importance to block transmission and allocate limited public health resources. According to the relationships among cases, we constructed disease transmission network graphs for the COVID-19 epidemic through a visualization technique based on individual reports of epidemiological data. We proposed an analysis strategy of the transmission network with the epidemiological data in Tianjin and Chengdu. The transmission networks showed different transmission characteristics. In Tianjin, an imported case of COVID-19 can produce an average of 2.9 secondary infections and ultimately produce as many as 4 generations of infections, with a maximum of 6 cases being generated before the imported case is identified. In Chengdu, 45 noninformative cases and 24 cases with vague exposure information made accurate information about the transmission network difficult to provide. The proposed analysis framework of visualized transmission networks can trace the transmission source and contacts, assess the current situation of transmission and prevention, and provide evidence for the global response and control of the COVID-19 pandemic.

## Introduction

In December 2019, an epidemic of viral pneumonia caused by a novel zoonotic coronavirus developed in Wuhan, the capital city of Hubei Province in China^1^. The World Health Organization (WHO) named the disease caused by the novel coronavirus “coronavirus disease 2019” (COVID-19) on February 11, 2020. COVID-19 often spreads by person-to-person transmission via respiratory droplets and close contact. The clinical features of COVID-19 are mainly fever, dry cough, and fatigue. However, some cases may progress to severe viral pneumonia with acute respiratory distress syndrome or even death^2^. The epidemic of COVID-19 spread rapidly to more than 200 countries^3^. The WHO has described COVID-19 as a pandemic due to the speed and scale of transmission^4^. By the end of January 2021, China had reported more than 100 thousand cases of COVID-19. Over 102 million infections and 2.2 million deaths have been confirmed worldwide since the start of the pandemic^3^.

Without effective population-based public health interventions, the rapidly increasing number of COVID-19 cases will greatly increase the burden of clinical treatments. At that point, the number of severe cases would exceed the capacity of the health system, which would result in a sharp rise in mortality^5–7^. However, due to the shortage of health resources caused by the global pandemic, it is difficult to implement large-scale screening or even censuses. Hence, identifying the transmission sources and pathways of COVID-19 is of vital importance to allocate limited public health resources. The integrated transmission chains and networks of COVID-19 transmission are revealed using epidemiological investigations. For example, based on the epidemiological data of 5830 confirmed cases in the early stage of the COVID-19 outbreak in Italy, researchers have constructed transmission chains through contact tracing. The results show that the outbreak started in the northern region of Italy as early as January 2020, rather than February 20, 2020, when the first COVID-19 case was confirmed in the Lombardy region^8^. In addition, close contacts indicated by transmission chains can also be used to identify potential susceptible groups, which can contribute to clarifying the emphasis of prevention, narrowing the scope and reducing the pressure of prevention and control, thus improving the efficiency of allocating limited health resources. Therefore, such comprehensive insight into the transmission network would be of great importance for local prevention and control policymakers.

Nevertheless, most current epidemiological investigation studies based on individual data primarily focus on describing the characteristics of COVID-19 infections, such as mortality, age distribution and sex ratio^9–12^. Information about exposure behaviors and contacts is normally provided by unstructured reports; it is heavily time-consuming to extract useful information from these reports to construct disease transmission networks. Additionally, such reports without a unified and structured form would be difficult to include for further analysis. To the best of our knowledge, no literature has visualized the relationship among COVID-19 infection cases using epidemiological data. In this study, the epidemiological data of each case, officially published from January 21 to February 22, 2020, in Chengdu and Tianjin, China, were used to construct visualized transmission networks according to the relationship among cases. Based on the constructed networks, the transmission characteristics of COVID-19 were visually presented and analyzed using measures of transmission networks. In addition, we explored the value of epidemiological investigation reports to provide evidence for the global response and control of the COVID-19 pandemic.

## Results

### Characteristics of COVID-19 infection

A total of 135 and 143 confirmed COVID-19 patients in Tianjin and Chengdu, respectively, were included. Four and 45 cases were noninformative cases in Tianjin and Chengdu, respectively. The 131 and 98 cases with valid information about exposure history in Tianjin and Chengdu, respectively, were used to construct transmission networks, of which 70 (53.43%) and 44 (44.89%) cases, respectively, were males. The median ages were both 49 years (ranging from 9 to 90 years in Tianjin and 3 months to 88 years in Chengdu). The median time from symptom onset to hospital admission was 2 and 3 days in Tianjin and Chengdu, respectively (Table 1). The median time from symptom onset to be defined as a confirmed case was 4.5 days in Tianjin and 6 days in Chengdu (Table 1).

**Table 1:** Characteristics of COVID-19 cases in Tianjin and Chengdu.

	Tianjin	Chengdu
Age, median (IQR), years	49 (36–61)	49 (33–59)
Male, *n*/*n*(%)	70/131 (53.43%)	44/98 (44.89%)
Time from symptom onset to be defined as a confirmed case, median (IQR), days*	4.5 (2–8)	6 (3–11)
Time from symptom onset to hospital admission, median (IQR), days*	2 (1–5)	3 (0–7)
Cases with exposure history, *n*/*n*(%)	131/135 (97.04%)	98/143 (68.53%)
Imported cases, *n*/*n*(%)	18/131 (13.74%)	30/98 (30.61%)
Nonimported cases, *n*/*n*(%)	113/131 (86.26%)	68/98 (69.39%)

* Four noninformative cases in Tianjin had information about sex and age, while 42 of the 45 noninformative cases in Chengdu had no individual information. To keep the statistical analysis consistent, the descriptions of all variables (except exposure history) were based only on the cases with valid information about exposure history.

*Of the 131 cases with an exposure history in Tianjin, 13 cases lacked information about key timelines (date of symptom onset, date of hospital admission, and date of confirmation as a case). Forty-four of 98 cases with an exposure history in Chengdu lacked information about key timelines. These cases were excluded only when the time from symptom onset to hospital admission was analyzed, as well as the time from symptom onset to confirmation as a case.

### Visualized transmission networks

**Figure 1:**
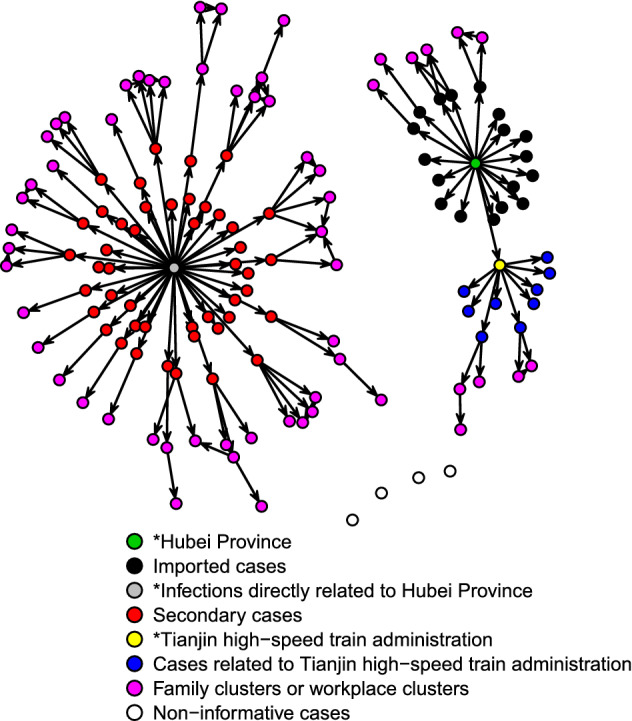
Transmission network graph for confirmed COVID-19 cases in Tianjin.

Figure 1 shows the COVID-19 transmission network graph in Tianjin. In the initial cluster of 18 (13.74%), cases had a history of exposure to Hubei Province, and each case infected an average of 2.9 contacts. In the component that started with the central node of infections directly related to Hubei Province, one case directly infected 0–4 contacts, with 0.79 as the average. At most, 6 patients were infected by one or more infection generations, with 5.68 as the average chain size for the initial cluster of imported cases. Twenty cases infected their relatives in the household or colleagues in the workplace, forming several multinode transmission chains with a maximum length of 4. Another 9 (6.87%) cases were employees of the Tianjin high-speed train administration. The transmission network of Tianjin consists of a total of 73 chains, of which 23 (36.99%) have a length greater than 1. There were 4 transmission chains with a maximum length of 4 in the component, starting with the central node of imported cases as the source of infection (Table 2).

**Table 2:** Distribution of transmission chains for COVID-19 cases in Tianjin and Chengdu.

Central node	Chain size	Tianjin		Chengdu	
		Maximum length of chains	Number of chains	Maximum length of chains	Number of chains
Hubei Province	1	1	13	1	29
	2	2	4	0	0
	3	3	1	2	1
Tianjin high-speed train administration	1	1	7	–	–
	3	3	1	–	–
	4	3	1	–	–
Infections directly related to Hubei Province	1	1	26	1	34
	2	2	7	2	3
	3	3	4	0	0
	4	4	4	0	0
	5	3	2	0	0
	6	4	3	0	0
Unclear exposures except Hubei Province	1	–	–	1	19
	3	–	–	3	1
	4	–	–	2	1
Total	–	–	73	–	88

**Figure 2:**
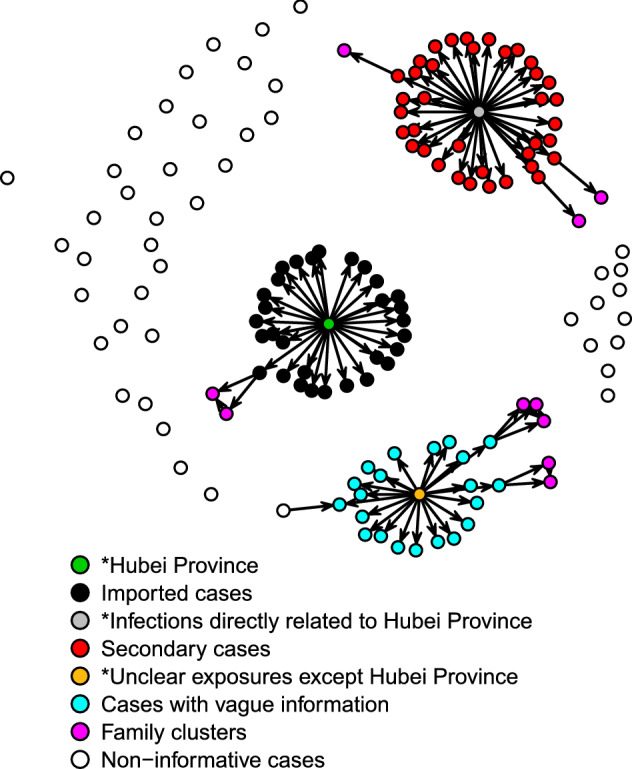
Transmission network graph for confirmed COVID-19 cases in Chengdu.

Figure 2 shows the transmission network graph of Chengdu. In the 98 (68.53%) informative cases in the transmission network, 30 (30.61%) cases had a history of exposure to Hubei Province. Each case generated at most 3 direct secondary infections and a maximum of 4 patients by one or more generations. In addition, for 24 cases with vague information, it was uncertain whether they were infected by contact with imported cases or by contact with other categories of cases. A total of 88 transmission chains were constructed in Chengdu. Twenty-one chains started from the central node of the unclear exposures except Hubei Province. Nineteen chains had a length of 1. The length of one chain was as high as 3 (Table 2).

### Applications of transmission network graphs

In this study, over 85% of patients in Tianjin were nonimported cases from the Tianjin high-speed train administration and multiple families. The longest length of the transmission chain of imported cases reached 4, which suggested that the spread of COVID-19 in Tianjin became dominated by community transmission. Not only strictly managing imported populations but also preventing community transmission was necessary to prevent the spread of COVID-19. In addition, the Tianjin high-speed train administration and related people should be a key target in further prevention and control measures. Similarly, the proportion of cases resulting from community transmission in Chengdu obviously exceeded that of the imported cases. Thirteen transmission chains in Tianjin with lengths greater than 3 suggested that these cases could spread for 3 generations before being confirmed. Therefore, the timeliness of case detection needs improvement. In contrast, the length of considerable transmission chains in Chengdu was lower than 3. However, 24 cases with vague information may be infected by two or more generations. One transmission chain with a length of 3 could be considered a chain with a length of as high as 6 if the starting node is an imported case. Therefore, considerable cases with vague information led to the fact that the length and number of transmission chains cannot accurately assess the spread of COVID-19.

There were only 4 noninformative cases in Tianjin, with a coverage rate of 97%. In Chengdu, as many as one-third of cases lacked exposure history. Compared with Chengdu, Tianjin had a higher quality of epidemiological investigations and a more complete transmission network. In Chengdu, the large number of noninformative cases and cases with vague information made it difficult to provide accurate information by the transmission network. Although the transmission chains in Chengdu were relatively short, many undiscovered and uncontrolled risks may exist that had not been revealed in the transmission network. Once community transmission accelerates, new transmission chains might rapidly appear and extend before cases are detected. Hence, in Chengdu, more healthcare workers need to be allocated to conduct epidemiological investigations to improve the coverage and quality of epidemiological investigations, the timeliness of detecting cases, and the rapidity of diagnosis.

## Discussion

Considering the relationships among cases, we constructed disease transmission networks and presented transmission network graphs for the COVID-19 epidemic through a visualization technique based on individual reports of epidemiological data. Then, in a framework of intuitive and quantitative analysis, we compared the transmission characteristics of COVID-19 in Tianjin and Chengdu in China. This valuable application of the visualization technique was further explored, including tracing the source of infections, discovering potential superspreaders, and evaluating prevention and control measures. Meanwhile, we discussed the potential insufficiency in the current form of individual epidemiological data. Our research may provide an important basis for jointly constructing multiregional and large-scale disease transmission networks.

Finding “patient zero” plays an important role in preventing the COVID-19 epidemic and studying the transmission characteristics^13–15^, as does the identification of the superspreading event^16–18^. However, in traditional epidemiological case reports, only the patient-related source of infections can be obtained from the collected exposure information, which cannot provide enough information to trace back to “patient zero” and present the full transmission chains. Thus, such reports need to be integrated using contact tracing analysis to construct transmission graphs to identify key nodes in transmission chains^19, 20^. For example, by contact tracing and constructing disease transmission chains, researchers found that the epidemic of COVID-19 in Italy had spread much earlier than February 20, 2020, when the first case was confirmed^8^. In our study, we found that one COVID-19 patient generally directly produced 0 to 4 infections in Tianjin, while in Chengdu, the number was as high as 3. There was no evidence of superspreaders in the two cities. Key nodes of the transmission chains can be applied to several aspects for prevention and control, including determining priorities and narrowing the scope of quarantine and thus allocating limited health resources effectively.

Currently, the available epidemiological data of each individual case vary from city to city. The exact exposure history can be extracted from released epidemiological data in Tianjin, while in Chengdu, only whether some of the cases have been in close contact with confirmed cases can be extracted, and the relationships among cases are not clear. From January 21 to February 22, 2020, 131 (97.04%) patients in Tianjin could be integrated into the transmission network, in which the source, relevant infections and terminal nodes could be revealed. In Chengdu, only 98 (68.53%) cases had infection pathways. The other 45 patients were noninformative cases, and thus, the sources and potential infectious ranges remain unclear. By comparing the disease transmission networks of the two cities, we found that the transmission network in Tianjin was more complete with clear transmission chains. However, in Chengdu, with a proportion of noninformative cases and cases with vague information, the tracing transmission chains of approximately one-third of the cases were not available, which suggests the risk of unclear transmission chains. Therefore, the number of nodes in each pathway and the close contacts of each node in the transmission network cannot be determined, indicating higher unpredictable risks in Chengdu. This result indicates that, in epidemiological investigations, the exposure history of each infected individual should be collected as completely as possible. Traceable transmission chains of each case would greatly reduce the unpredictable risks and avoid the waste of health resources. In addition, the composition of the transmission chains presents the main type of local transmission, which suggests that further prevention and control should focus more on imported or community-spread cases. The length of transmission chains partly suggests that the timeliness of case detection, as well as the quality of the epidemiological investigation reflected by the rate of case coverage, provides a valuable index for evaluation of the efficiency of the local control measures. The relatively poor quality of epidemiological data in Chengdu may suggest a shortage of public health manpower, which may provide evidence for adjusting control and prevention strategies and allocating resources.

Due to various forms of local epidemiological investigation reports, it is difficult to construct an integrated cross-regional transmission network and gain full use of the epidemiological data for COVID-19 prevention and control. Thus, we suggest that health administrations develop a standard guideline for epidemiological data collection, and all such data should be managed and released in a timely manner^21^. On the one hand, researchers can jointly construct a multiregional transmission network to trace the spread of COVID-19. On the other hand, integrated transmission networks can improve public awareness of COVID-19 epidemics, enhance public compliance with control measures, and reduce the difficulty of implementation and resource consumption. Moreover, with transmission networks, network-based analysis can be performed to evaluate the transmission rates and the complexity of network structures, which may provide clues for large-scale interventions. In addition, for emerging infectious diseases, constructing transmission chains through contact tracing can estimate infectivity at an early stage to quantify the risk and trends of infectious diseases^22–24^.

Currently, there are two main situations of the epidemic with different prevention and control priorities. On the one hand, in countries with relatively stable situations, the primary challenge is to discover and control sporadic cases, clusters of cases, and potential community transmission. The proportion of the susceptible population is far greater than that of infections. Tracing COVID-19 cases seems a resource-saving method in these countries. Therefore, the proposed analysis of visualized transmission networks provides a helpful tool for many countries, especially those in the early stage of the epidemic or facing a new wave coming with the winter. On the other hand, in several other regions, it is too late to stop transmission chains without lockdown among the whole population. Individual epidemiological data collection and network analysis could be time- and human resource-consuming. An alternative approach for these regions is the analysis of real-time data in a determinate environment, such as prisons and factories, in which the data of individual tracks are easy to collect and privacy issues are generally not considerable^25^. However, transmission networks could still provide practical information in assessments of epidemics and improvements in control and prevention measures.

It is worth noting that COVID-19 cases in one category might be included in another category in transmission network graphs. For instance, some cases in the category of family clusters are contained in the category of exposure to infections directly related to Hubei Province. To fully demonstrate the information contained in the epidemiological data, however, this study classified these patients into another category, with family aggregation as a vital feature of infectious disease transmission. Better classification strategies are needed to discuss the local transmission characteristics in different cities. Meanwhile, regional unification should be considered for the classification standard to guarantee the exchangeability of data when cross-regional transmission networks are constructed. In addition, new technological approaches such as smartphone applications, artificial intelligence, and geolocalization have been harnessed to support the public health response to COVID-19^26–29^. For instance, smartphone applications used in contact investigations of COVID-19 can supplement more objective evidence of patients’ trajectories. If the new technological approaches could be used together with the analysis of network visualizations, it will be more convenient to construct a large-scale, multicenter COVID-19 transmission network, which may provide more information for identifying key areas and launching a multiregional public health response.

For infectious diseases with stronger infectivity, longer incubation periods and higher fatality risks, such as COVID-19, the number of cases would increase rapidly, consume limited clinical resources quickly and lead to high mortality, if public health interventions were not conducted in a timely and effective manner^30^. Therefore, the emphasis of control measures should not only focus on clinical treatments but also ensure sufficient resources in epidemiological investigations^31^. The epidemiological data of the COVID-19 epidemic in Tianjin and Chengdu were used to propose an analysis framework for the individual epidemiological data. Our results illustrated the importance of visualized epidemiological transmission networks in preventing and controlling the epidemic of COVID-19. Currently, the content and format of epidemiological data are not unified, causing the transmission network graphs of Tianjin and Chengdu to show different performances in risk assessment. Therefore, the collection, management, and release of epidemiological data should be improved for the joint construction of large-scale and multiregional disease transmission networks to provide a better understanding of the COVID-19 epidemic and to provide evidence for local prevention and control policymakers.

## Methods

### Data collection and information extraction

Since January 21, 2020, the official websites of several municipal health commissions in mainland China have been continuously releasing individual records of daily confirmed COVID-19 cases. As of February 22, 23 (74.19%) of the 31 capitals/municipalities in mainland China had begun to publish individual reports, most of which were unstructured reports with different forms and content. Detailed relationships among individual cases, such as relatives, colleagues, or other contacts, can be obtained in some cities, such as Tianjin, Chongqing and Xinyang. In other cities, such as Chengdu, Beijing, and Shanghai, only limited exposure information can be extracted for a few cases. For instance, some cases in Chengdu were reported to be related to other confirmed cases, but no detailed information was available to indicate which specific confirmed cases were related. Daily individual records of the confirmed COVID-19 cases in Tianjin and Chengdu from January 21 to February 22 were used in our analysis; these data were collected from the websites of municipal health commissions.

**Figure 3:**
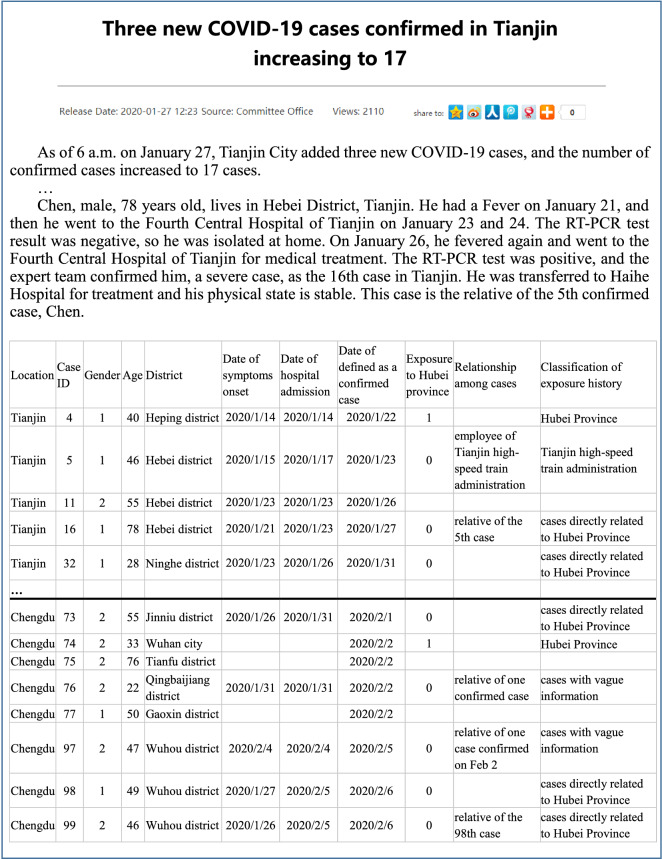
Example of unstructured individual reports and structured databases of Tianjin and Chengdu.

Information was extracted from the individual records to build a structured database including 4 sections: demographic characteristics (sex, age, and district); key timelines (date of symptom onset, date of hospital admission and date of confirmation as a case); exposure history (exposure to Hubei Province and relationship among cases); and classification of exposure history^32^. There were two common categories of exposure history in cities: exposure to Hubei Province and exposure to cases directly related to Hubei Province. The other identical characteristics among exposure histories of several cases were extracted to new categories. The exposure histories of the clustering cases who worked in Tianjin high-speed train administration were classified as exposed to Tianjin high-speed train administration. The exposure histories of the cases in Chengdu with a history of exposure to confirmed cases but without detailed information to identify the specific related cases were classified as being exposed to cases with vague information. For example, one new case (patient 97) in Chengdu confirmed on February 9 was reported to be relevant to another case confirmed on February 2. However, on February 2, 4 cases were confirmed. It is unclear that which one of the 4 cases was related to patient 97. Cases without exposure history were defined as noninformative cases. We provide an example of the unstructured individual reports and the structured individual line list in Fig. 3^33^.

### Construction of transmission networks

The transmission networks were built with three key elements: central nodes representing categories of exposure history, nodes representing confirmed cases and directional edges. We constructed the transmission networks in the following two steps.

First, the central nodes were set based on the categories of exposure history.

Three central nodes were set to represent the sources of exposure. Hubei Province, infections directly related to Hubei Province, and the Tianjin high-speed train administration were set as the starting central nodes of the transmission network in Tianjin. Similarly, for the transmission network of Chengdu, three central nodes were set to present Hubei Province, infections directly related to Hubei Province, and unclear exposures except for those in Hubei Province.

Then, cases were integrated as nodes into the transmission networks by the source of exposure.

In the transmission networks, the nodes other than the starting nodes represented confirmed cases. Those cases that had clear contact histories with specific confirmed cases were linked with directional edges. The directions of edges denoted the direction of COVID-19 transmission between cases. The cases without related cases in the exposure history were directly linked to the corresponding central node of the source of exposure. Nodes of noninformative cases without the exposure history scattered outside the transmission network and were not part of the components in the transmission network. Starting with the nodes of the earliest traceable sources of infection, the transmission chain was composed of one central node, corresponding directional edges, and all nodes of secondary cases were linked by one or more generations of transmission.

### Quantitative characteristics of transmission networks

**Figure 4:**
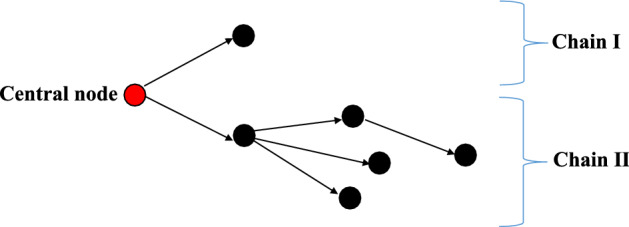
Example of a simplified transmission network with a group of 2 cases as the central node and a total of 2 chains in the network.

Different characteristics were described by quantitative measures of the transmission networks, i.e., the number of chains, chain sizes, maximum lengths of chains, average chain size and average number of secondary nodes linked to the nodes in the previous generation of cases. The number of chains, chain sizes and average chain size were used to indicate the scope of COVID-19 through one source of infection. The maximum lengths of chains and average number of secondary nodes linked to the nodes in the previous generation of cases were calculated to present transmission scales. The detailed definitions and implications of these indexes in COVID-19 are shown in Table 3 and illustrated using a simplified sample in Fig. 4.

**Table 3:** The detailed description of 5 indexes applied for assessing the evolving epidemiology of COVID-19.

Index	Definition	Implication in COVID-19	Example in Fig. 4
Number of chains	The number of chains starting with a central node	The spread of transmission through the source of infection	2
Chain size	The number of nodes in each transmission chain except the central node	The number of cases in a chain and the scope of a transmission chain of COVID-19	Chain I: 1 Chain II: 5
Maximum length of chains	The maximum number of directional edges in each chain	The maximum generations of transmission before the secondary case is detected and controlled	Chain I: 1 Chain II: 3
Average chain size	Dividing the summation of chain sizes starting with same central node by the number of cases in the central node	The average reproductive number of cases from specific exposures	6/2 = 3
Average number of secondary nodes linked to the nodes in the previous generation of cases	Dividing the total number of nodes in the same distance with central node by the total number of front-end nodes	The infectivity of different generations of cases	First generation: 2/2 = 1 Second generation: 3/2 = 1.5 Third generation: 1/3 = 0.3

These indexes were then summarized to compare the transmission characteristics in Tianjin and Chengdu. The average chain size and number of nodes linked to each generation of cases were quantified only in Tianjin’s transmission network, as in Chengdu, approximately one-third of the confirmed cases of COVID-19 cannot be integrated into the transmission network due to uncertain exposure history.

All the transmission networks were constructed and visualized with R3.5.1 using the package *statnet*.

### Ethics statement

All patient information on COVID-19 epidemiological data was collected from the official websites of municipal health commissions, and this study was approved by the institutional review board of the School of Public Health, Sichuan University. All methods were conducted in accordance with relevant guidelines and regulations. All data were collected from publicly available sources. Data were deidentified, and informed consent was waived.

## Data Availability

The datasets used and/or analyzed during the current study are available from the corresponding author on reasonable request.
